# Only as strong as the weakest link: structural analysis of the combined effects of elevated temperature and pCO_2_ on mussel attachment

**DOI:** 10.1093/conphys/coz068

**Published:** 2019-10-31

**Authors:** Laura A Newcomb, Matthew N George, Michael J O’Donnell, Emily Carrington

**Affiliations:** 1 Department of Biology, Life Sciences Building, University of Washington, Box 351800, Seattle, WA 98195, USA; 2 Friday Harbor Laboratories, University of Washington, 620 University Road, Friday Harbor, WA 98250, USA; 3 Department of Bioengineering, 306 Stanley Hall #1762, University of California, Berkeley, CA 94720, USA

**Keywords:** Byssal thread, ecomechanics, multiple stressors, *Mytilus*, ocean acidification, ocean warming

## Abstract

Predicting how combinations of stressors will affect failure risk is a key challenge for the field of ecomechanics and, more generally, ecophysiology. Environmental conditions often influence the manufacture and durability of biomaterials, inducing structural failure that potentially compromises organismal reproduction, growth, and survival. Species known for tight linkages between structural integrity and survival include bivalve mussels, which produce numerous byssal threads to attach to hard substrate. Among the current environmental threats to marine organisms are ocean warming and acidification. Elevated pCO_2_ exposure is known to weaken byssal threads by compromising the strength of the adhesive plaque. This study uses structural analysis to evaluate how an additional stressor, elevated temperature, influences byssal thread quality and production. Mussels (*Mytilus trossulus*) were placed in controlled temperature and pCO_2_ treatments, and then, newly produced threads were counted and pulled to failure to determine byssus strength. The effects of elevated temperature on mussel attachment were dramatic; mussels produced 60% weaker and 65% fewer threads at 25°C in comparison to 10°C. These effects combine to weaken overall attachment by 64–88% at 25°C. The magnitude of the effect of pCO_2_ on thread strength was substantially lower than that of temperature and, contrary to our expectations, positive at high pCO_2_ exposure. Failure mode analysis localized the effect of temperature to the proximal region of the thread, whereas pCO_2_ affected only the adhesive plaques. The two stressors therefore act independently, and because their respective target regions are interconnected (resisting tension in series), their combined effects on thread strength are exactly equal to the effect of the strongest stressor. Altogether, these results show that mussels, and the coastal communities they support, may be more vulnerable to the negative effects of ocean warming than ocean acidification.

Now in building of chaises, I tell you what,

There is always somewhere a weakest spot.

“The One Hoss Shay” by Oliver Wendell Holmes (1858).

## Introduction

Organisms manufacture and maintain complex structures that provide a broad range of mechanical functions. A few examples include vertebrate musculoskeletal systems for locomotion, spider webs for prey capture, hard shells for predator deterrence, and plant roots and algal holdfasts for anchorage (Wainwright *et al*., 1976; Vogel, 1988; Denny, 2016). Structural failure can compromise an organism’s reproduction, growth, and survival, motivating the development of biomechanical frameworks to estimate the risk of failure, such as dislodgment and breakage in benthic marine organisms (Johnson and Koehl, 1994; Denny, 1995; Koehl, 1999; Madin, 2005; Madin *et al*., 2012; Carrington *et al*., 2009, 2015) and root and stem lodging in crop plants and trees (Baker *et al*., 1998; Berry *et al*., 2004; Spatz and Bruechert, 2000). A common feature of these ‘ecomechanical’ frameworks is to compare environmental loading to the organism’s strength, often with feedback incorporated from various organism–environment interactions.

Interest in the effect of environmental stressors on marine organisms has intensified because ocean conditions are changing at rates unprecedented in geological history and are projected to have wide-scale effects (Doney, 2010; Kroeker *et al*., 2013; Gunderson *et al*., 2016). Stressors, such as ocean warming and acidification (increased pCO_2_), have been shown to weaken the skeletal elements, shells, and adhesives marine organisms build to persist in their environment (Collard *et al*., 2016; Gaylord *et al*., 2011; Madin *et al*., 2012; Newcomb *et al*., 2015; Guenther *et al*., 2018). As with many other physiological responses, these effects are often nonlinear and context dependent, complicating the development of predictive frameworks to assess the combined effects of multiple stressors (Ivanina *et al*., 2013; Gaylord *et al*., 2015; Gunderson *et al*., 2016; Kroeker *et al*., 2017; Boyd *et al*., 2017).

**Figure 1:**
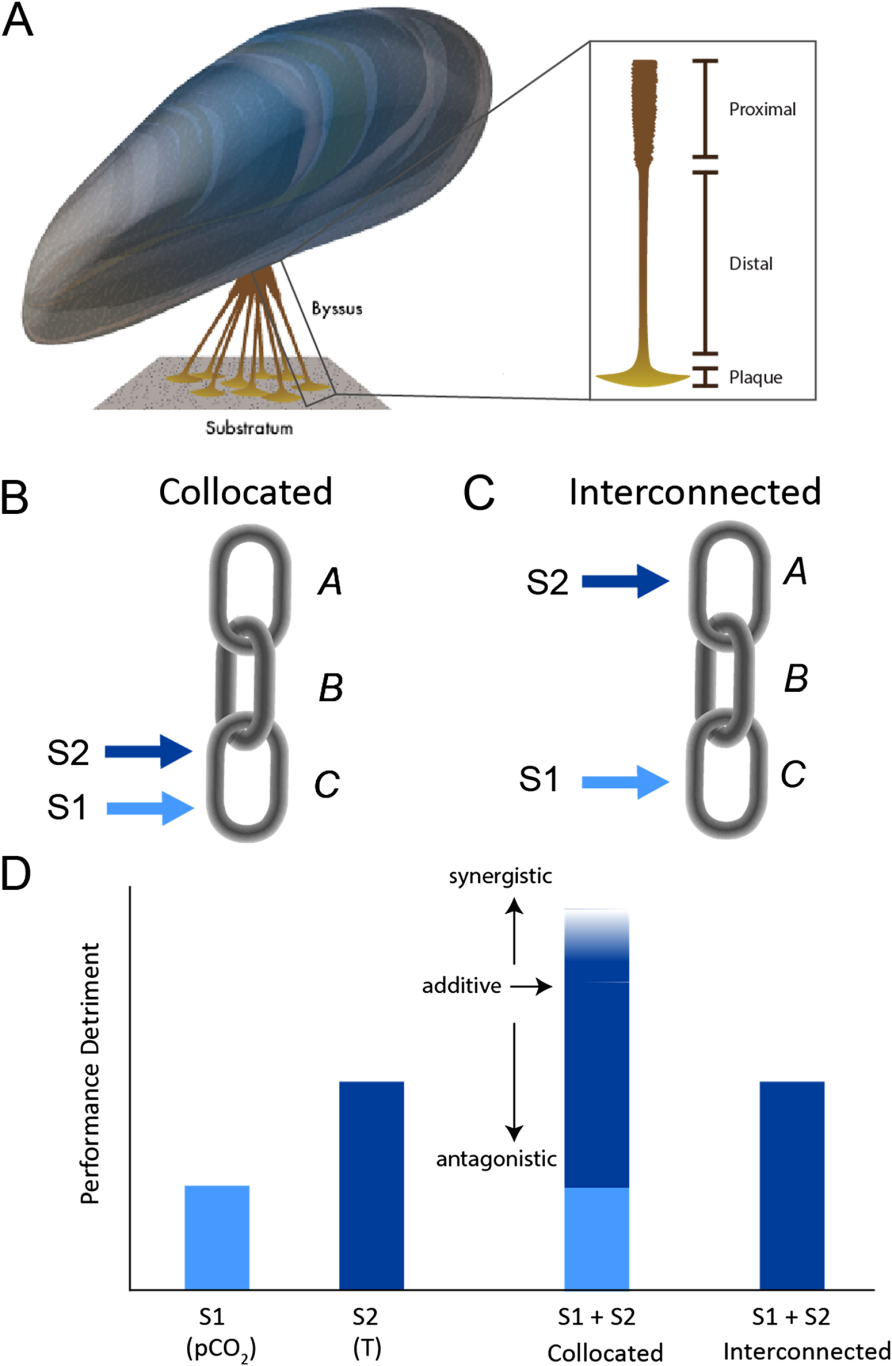
A conceptual model of the potential effects combined stressors on mussel byssal attachment, adapted from the more general ecophysiological model of Gunderson *et al*. (2016). (A) A mussel tethers itself to substrate with numerous byssal threads, each comprising three morphologically and functionally distinct regions (proximal, distal, and adhesive plaque) that are connected in series, similar to links in a chain. Assume two stressors, S1 and S2, each act to weaken a byssal thread in a specific region (link). If the two stressors are collocated and target the same region (B), they may produce synergistic, additive, or antagonistic interactions that are greater, than equal to, or less than, respectively, the sum of their individual effects on thread performance (D). If the stressors instead target different but interconnected regions (C), thread performance is influenced only by the larger detrimental effect; the stressor that creates the weakest link masks the effect of the other stressor on another region in the chain. In the configuration shown, with S1 < S2, the failure location is in link C for stressors that are collocated, but in link A for stressors that are interconnected.

The complexity of the effects of multiple environmental stressors on a biomechanical structure is evident in marine mussels, including several ecologically and economically important species. Mussels produce an array of byssal threads to tether themselves to hard substrate, providing protection from dislodgment by predators, waves, and currents (Fig. 1; Carrington *et al*., 2015). Mussel attachment strength is the product of byssal thread number and quality, both of which have been shown to vary with environmental conditions. For example, elevated pCO_2_ (>1200 μatm) lowers thread strength in *Mytilus trossulus* (O'Donnell *et al*., 2013; George and Carrington, 2018), thread strength and production in *Mytilus coruscus* and *Pinctada fucata* (Zhao *et al*., 2017; Li *et al*., 2017; Kong *et al*., 2019), and thread strength in *Mytilus edulis* (Kong *et al*., 2019, but see Dickey *et al*., 2018). However, there is little consensus when whole animal attachment is measured, with negative, neutral, and positive effects of elevated pCO_2_ observed in *Mytilus galloprovincialis*, *M. edulis, Xenostrobus securis*, and *M. coruscus*, respectively (Babarro *et al*., 2018; Clements *et al*., 2018; Sui *et al*., 2017). These same studies reported negative effects of elevated temperature on attachment in *M. galloprovincialis* and *M. edulis*, but not *X. securis*. Moreover, the negative effect of temperature on attachment was dependent on the level of pCO_2_ treatment in *M. galloprovincialis*, but not *M. edulis*. While this broad variation in the observed effects of elevated pCO_2_ and temperature on byssus attachment in part reflects species-specific responses (Babarro *et al*., 2018; Clements *et al*., 2018), we propose that a structural analysis approach, one that evaluates a system by examining its component parts and their interactions provides an improved framework for assessing the effects of multiple stressors on complex biological support structures. In this study, we use this approach to evaluate the dependence of mussel attachment strength on two stressors, elevated pCO_2_ and temperature, applied in isolation and combination.

Mussel byssal threads are multi-component tensile structures, comprising three serially linked regions that differ in material composition and mechanical function (Fig 1a; Carrington *et al*., 2015). Similar to the components of the skeleton of a leg or the oxygen transport system in mammals (Alexander, 1997), each region of the thread can be represented as a link in a chain; the strength of the thread (a higher order structure) is determined by the weakest link. For mytilid mussels, the weakest link is typically the proximal or plaque region (Bell and Gosline, 1996; Carrington *et al*., 2015). Each thread region is distinct in its morphology, ultrastructure, and chemical composition (see reviews by Carrington *et al*., 2015; Waite, 2017); thus, it is unlikely that a given environmental stressor affects each region in exactly the same way. Indeed, both O'Donnell *et al*. (2013) and Zhao *et al*. (2017) found that the negative effect of increased pCO_2_ was localized to the adhesive plaque, biasing the failure mode to this region.

Using the chain analogy for a byssal thread (Fig 1b), we represent this localized effect of a given stressor (S1) on a byssus component (link C). If a second stressor (S2) is collocated and targets the same region, then there is the potential for direct interaction between the two stressors. As reviewed by Gunderson *et al*. (2016), the interaction is considered to be additive if the combined stressor effect on thread strength is equal to the sum of the individual effects (Fig 1d). An interaction that is non-additive and results in a combined effect that is greater than or less than the sum of the individual effects is termed synergistic or antagonistic, respectively. However, if S2 targets a different link in the chain (e.g. link A, Fig 1c), the two stressors act independently on interconnected components and only the stressor with the strongest detrimental effect (the one that creates the weakest link) will determine the size of the combined effect on thread strength (Fig. 1d). Statistically (e.g. using a general linear model), this is a special case of an antagonistic interaction where the combined effect size is exactly equal that of the greater stressor. The effect of the lesser stressor is only ‘observable’ when the greater stressor is not applied. In this manner, knowledge of the stressor effect size, as well as the target location, is critical to predicting the performance of the entire chain in the presence of two stressors.

Scaling up, whole-mussel byssal attachment can be represented by numerous chains that bear load in parallel, and the number, strength, and compliance of these chains determine overall strength of the byssus structure (Bell and Gosline, 1996). A stressor that affects the number of chains present, therefore, has the potential to exacerbate or compensate for any detrimental effects on the individual chain components.

We used this structural analysis framework to evaluate the relative effect of two environmental stressors on mussel byssal attachment strength. We exposed mussels (*M. trossulus*) to a range of environmentally relevant temperature and pCO_2_ conditions and quantified the effects on the material properties of each byssal thread region, the whole byssal thread, and the number of threads produced. These thread-level effects were then integrated into a model estimating whole-mussel attachment strength. We find (i) elevated temperature dramatically weakens byssal thread strength and production, and these effects combine to nearly decimate overall mussel attachment; (ii) elevated temperature and pCO_2_ affect different components of byssal threads; and (iii) the effect of pCO_2_ was variable, ranging from neutral to slightly positive, and was masked by the stronger negative effects of elevated temperature.

## Materials and Methods

Mussels (*M. trossulus*) were collected in May 2012 from Argyle Creek, San Juan Island, WA (48.52° N, 123.01° W) and held in a mesh box submerged under the dock at Friday Harbor Laboratories (FHL), San Juan Island, WA, for up to 14 days. Mussels were placed in experimental mesocosms in the Ocean Acidification Experimental Laboratory at FHL as described in O'Donnell *et al*. (2013) and Timmins-Schiffman *et al*. (2013). Briefly, manipulations of pH were made by bubbling CO_2_ into a 150 L of temperature-controlled seawater reservoir, which supplied water to eight 3.5 L of chambers at a turnover rate of 50 mL min^−1^. Air was bubbled into the reservoir to maintain 100% oxygen saturation and submersible pumps (Model Number P396; Annex Depot, Sacramento, CA, USA) provided mixing in the chambers at 3.8 L min^−1^. The bottom of each chamber was lined with autoclaved pebbles, collected from an FHL beach, to provide a substrate for byssal thread attachment. pH and temperature were monitored continuously in each water reservoir with a Durafet pH and temperature probe and the full carbonate chemistry of the system evaluated with dissolved inorganic carbon (DIC) and total alkalinity measurements once during each trial. Mussels were acclimated to their treatment temperatures in ambient pH (~7.8) over 9 days, ramping temperature up no more than 2°C per day, and fed a maintenance level of Shellfish Diet 1800 (6 g l^−1^ day^−1^; Reed Mariculture, Campell, CA, USA).

The 12 independent temperature × pCO_2_ treatments spanned the range of local marine conditions (Newcomb, 2015; George *et al*., 2019; temperature at 10°C, 18°C, or 25°C and pCO_2_ at 400, 750, 1200, or 2500 μatm). Each mussel was trimmed of external byssus before placement in an experimental treatment for 3 days, sufficient time to produce new mature byssal threads (Bell & Gosline, 1996) while minimizing the effect of treatment on mussel condition. Mussels were starved during the 3-day trials to minimize changes in chamber water chemistry due to food addition and to reduce fouling. Three trials were conducted in succession to replicate treatments over time, increasing sample size (*n* = 8 × 3) for each temperature × pCO_2_ treatment.

At the end of each trial, mussels and the rocks to which they had attached with byssal threads were removed from the chambers. The entire byssus was dissected from each mussel and stored air dried for up to 20 days. Byssus was rehydrated in seawater prior to testing, a method that does not alter the mechanical properties of the byssal threads (Brazee, 2004). The number of byssal threads each mussel produced was counted, and one thread was haphazardly chosen for mechanical testing following the procedure of Bell & Gosline (1996). Briefly, an individual thread was clamped with submersible pneumatic grips on either end by holding the proximal byssal stem between cardstock with cyanoacrylate glue and affixing the distal plaque with attached rock to an aluminum T-bar with epoxy. An Instron 5565 tensometer (Norwood MA, USA) extended the thread at a rate of 10 mm min^−1^ in a temperature-controlled water bath (3130-100 BioPuls Bath; Instron, Norwood, MA, USA) until failure. The tensometer measured force (±10^−3^ N) and extension (± 10^−3^ mm) at 10 Hz. Tests were performed in seawater with a pH of 7.8 and the relevant treatment temperature.

Pull to failure mechanical tests provided estimates of thread breaking force, yield force, extensibility, initial stiffness, and failure location (Bell & Gosline, 1996). Yield, due to quasi-plastic deformation in the distal region, was identified as the point where the initial slope of the force extension curve decreased by 40%. Extensibility was calculated by dividing thread extension at failure by initial length, and initial stiffness was determined from the initial slope of the force extension curve. The location of failure (proximal, plaque, and/or distal region) was noted, and threads were retested to quantify the breaking force of each remaining region. Tests that broke at the grips were considered underestimates and were discarded.

The cross-sectional area of the proximal region was measured to evaluate morphological differences among treatments. The elliptical area was estimated from measures of the major and minor axes (± 1 μm using a dissecting microscope) (Brazee & Carrington, 2006). Proximal breaking stress (N mm^−2^), a material property, was calculated as proximal breaking force divided by proximal area. Thread surface structure was examined using a scanning electron microscope (FEI Sirion XL30 SEM, Hillsboro, OR, USA).

Whole-mussel attachment strength was estimated using two mathematical models developed by Bell & Gosline (1996). Each model assumes a mussel is anchored with a constant thread number (*n* = 50) arranged in a circle. The normal model estimates dislodgment force perpendicular to the substrate (e.g. lift); all threads are engaged and extend until they reach their maximum force. The parallel model estimates dislodgement force for an animal pulled parallel to the substrate (e.g. drag); threads on the upstream side are the first in tension, yield, and extend until they reach maximum force and break, while more threads are recruited into tension until they have all broken. Additionally, we modified each model to incorporate the variation in thread production across treatments. Because thread production was measured for only 3 days, treatment means were scaled to a maximum value of 50 threads.

Statistical tests were carried out in R version 2.13.0 (R Development Core Team, Vienna, Austria, 2011, packages: plotrix, RStudio, sciplot). Thread breaking force (whole, proximal, plaque), distal yield force, and extensibility were square root transformed to meet the assumptions of normality. These response variables, in addition to thread production, were analyzed with linear mixed-effect models (lmem) with pCO_2_ and temperature as fixed factors and trial as a random factor. Proximal region long axis, short axis, and breaking stress were compared among temperature treatments with lmem with temperature as a fixed factor and trial as a random factor. The effect of treatment on failure location was tested with a Pearson’s χ^2^ test. The effect of temperature on the difference between proximal and plaque strength in an individual thread was tested with a Kruskal–Wallis test and a Tukey’s HSD post-hoc test detected differences between treatments. The effect of treatment was considered significant with an α ≤ 0.05.

## Results

Treatments reached within 5% of their targeted temperature, while pCO_2_ for replicate treatment conditions varied 1–32% from the mean of all trials (Supplementary Table S1). The random factor of trial did not significantly affect any of the response variables (Table 1).

**Table 1:** Summary of statistical analyses of the effects of elevated temperature and pCO_2_ on byssal thread mechanics and production. Linear mixed-effect models were run with pCO_2_ and temperature as fixed factors and trial as a random factor on each of the the following dependent variables: whole thread strength, proximal strength, plaque strength, and thread number. Temperature and pCO_2_ × temperature models were compared to the null model of pCO_2_ (see Table S1 for N)

			**Whole Thread Strength (N)**	**Proximal Strength (N)**	**Plaque Strength (N)**	**Thread Number**
Random effect of trial		SD	0.000	0.000	0.000	1.58
ANOVA on lmem	Temperature	χ^2^	93.6	87.9	34.7	61.1
		df	2	2	2	2
		*P*	**<0.001**	**<0.001**	**<0.001**	**<0.001**
	pCO_2_	χ^2^	3.8	1.7	10.2	13.5
		df	3	3	3	3
		*P*	0.3	0.6	**< 0.05**	**< 0.01**
	Temperature × pCO_2_	χ^2^	4.6	1.1	8.2	7.7
		df	6	6	6	6
		*P*	0.6	0.9	0.2	0.3
Tukey HSD	Temperature	pCO_2_				
	10	400	a	a	a	a
	10	750	a	a	ab	ab
	10	1200	a	a	a	a
	10	2500	a	a	b	abc
	18	400	a	a	a	a
	18	750	a	a	ab	abc
	18	1200	a	a	a	a
	18	2500	a	a	b	a
	25	400	b	b	c	bc
	25	750	b	b	cd	c
	25	1200	b	b	c	bc
	25	2500	b	b	d	c

ANOVA is analysis of variance. HSD is honestly significant difference

**Figure 2:**
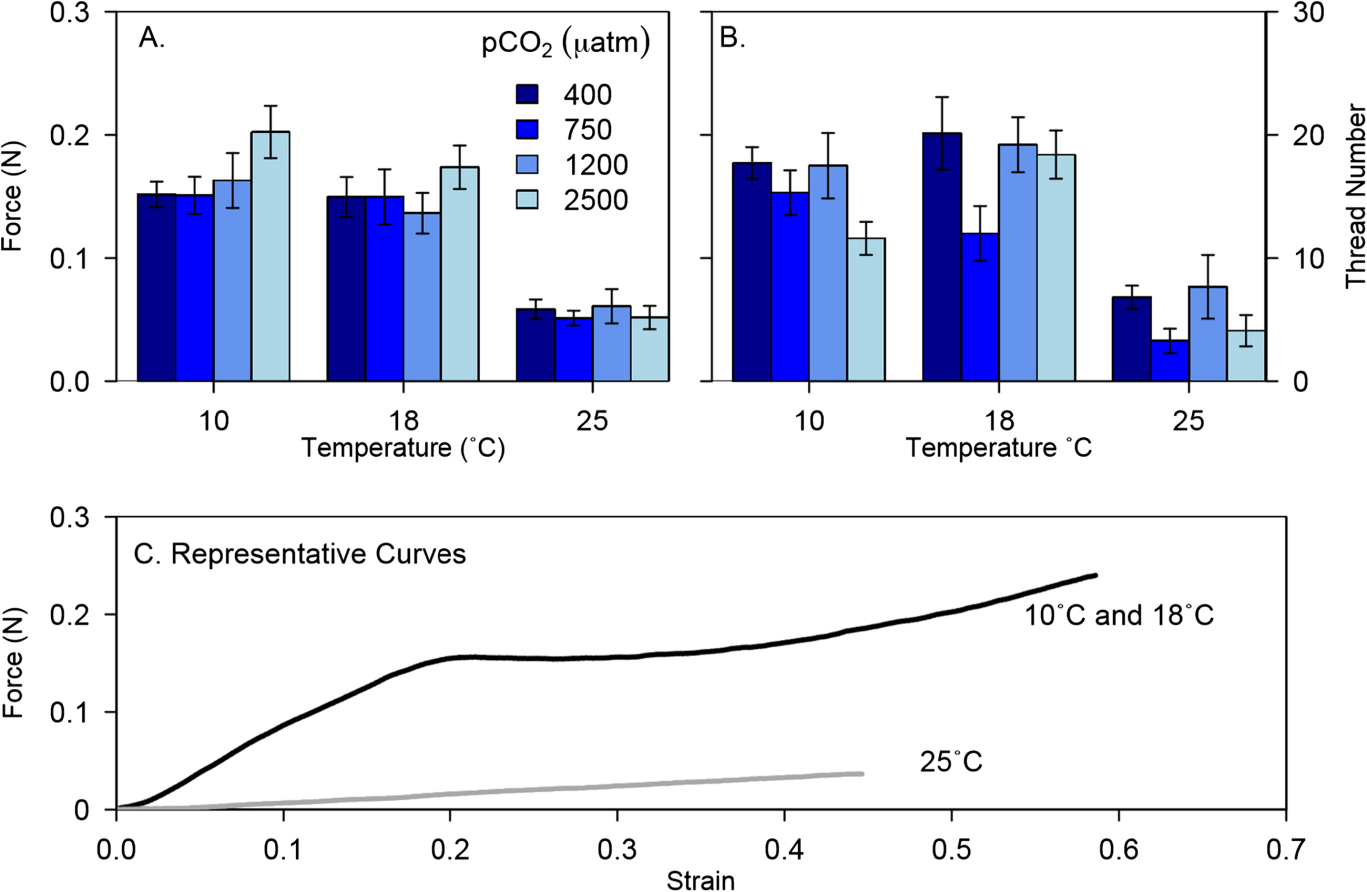
Whole thread breaking force (A) and thread production (B) as a function of seawater temperature and pCO_2_ (μatm). At 25°C, byssal thread strength was significantly weaker and production was significantly slower (lmem, *P* < 0.001), while the effect of pCO_2_ was not significant (see Table 1 for statistical summary). Symbols are means ± SEM; N reported in Supplementary Table S1. (C) Representative force versus extension curve of a mussel byssal thread at high (25°C) and low (10°C or 18°C) temperature. A low temperature thread is initially stiff, extends and yields in the distal region, and then stiffens until it breaks in either the plaque or proximal region. Threads formed at high temperature (25°C) are less stiff, do not yield, and are weaker and less extensible.

Byssal thread breaking force was affected by temperature, but not pCO_2_ (Fig. 2, Table 1, lmem, *P* < 0.001 and *P* = 0.3, respectively); threads produced at 25°C were 60% weaker than those produced at 10°C and 18°C. Mussels grown at 25°C produced 65% fewer threads than those in 10°C and 18°C (Fig. 2; Table 1, lmem, *P* < 0.001). There was a significant effect of pCO_2_ (lmem, *P* < 0.01), but no clear pattern among combinations of treatments was evident and there was no interaction between the two factors (Table 1, lmem, *P* = 0.3).

**Figure 3:**
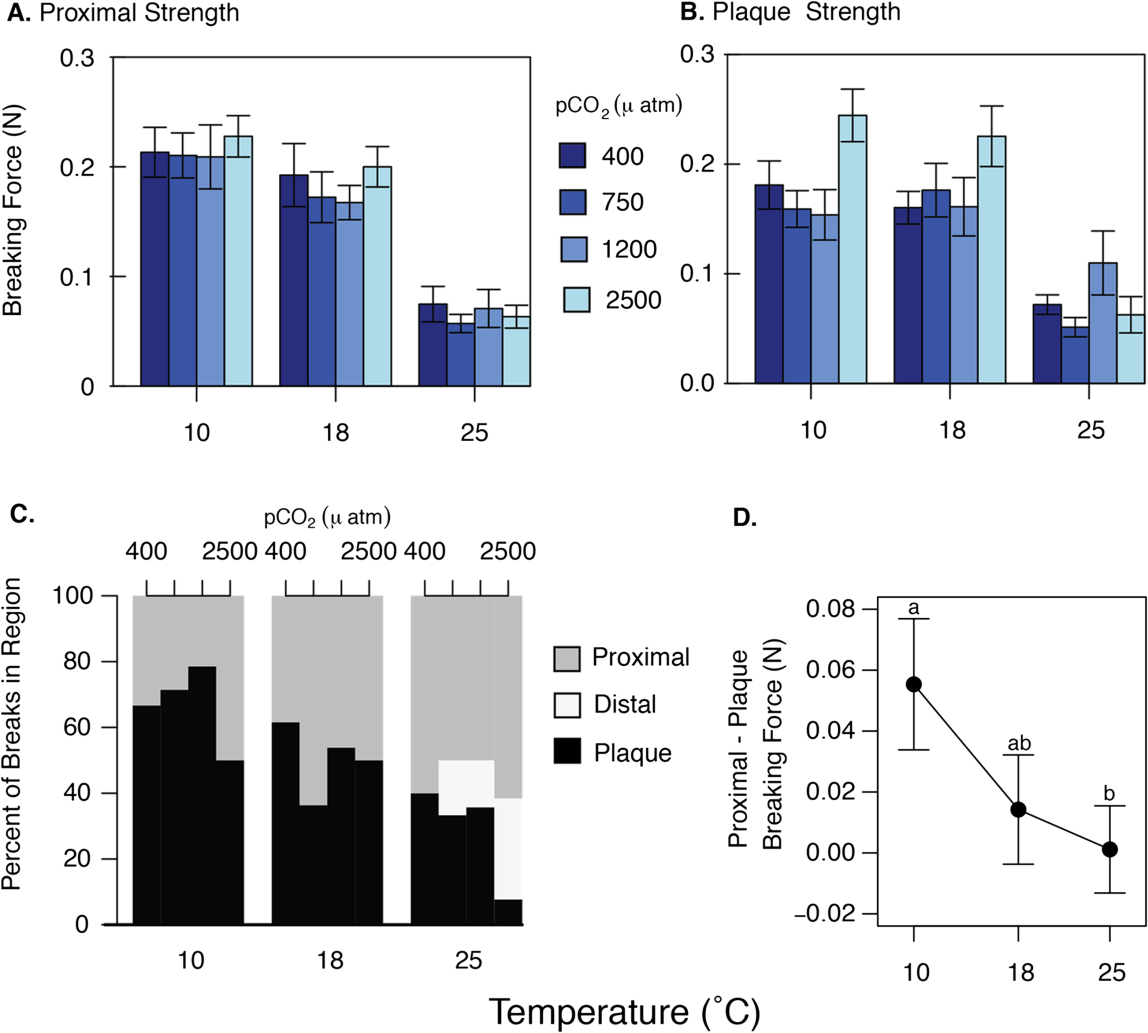
Summary of tensile mechanical testing for each region of the byssal threads produced in the experimental treatments. Proximal (A) and plaque (B) region breaking force (N) as a function of seawater temperature and pCO_2_ (μatm). Both regions were significantly weaker at 25°C (lmem, *P* < 0.001). The plaque was the only region sensitive to pCO_2_, with a weak positive response (lmem, *P* < 0.05). Bars are means ± SEM; see Table 1 for statistical summary and Supplementary Table S1 for N for each treatment. (C) Percentage of thread failures in each region for each temperature and pCO_2_ treatment. The location of failure shifted toward the proximal region with increased temperature but was not affected by pCO_2_ (Pearson’s χ^2^, *P* < 0.05 and 0.3, respectively). Distal breaks were infrequent and only observed in the 25°C treatments. (D) As temperature increased, the difference between proximal and plaque breaking force decreased significantly (Kruskal–Wallis, *P* < 0.05), thereby decreasing the likelihood of failure in the adhesive plaque.

The breaking strength of the proximal region was 70% weaker at 25°C in comparison to 10°C and 18°C (Fig. 3A, Table 1, lmem, *P* < 0.001), but the effects of pCO_2_ and its interaction with temperature were not significant (Table 1, *P* = 0.6 and 0.9, respectively). The adhesive plaques produced at 25°C were 60% weaker than those produced at 10°C and 18°C (Fig. 3B, Table 1, lmem, *P* < 0.001), but unlike the other breaking force metrics, elevated pCO_2_ increased plaque strength; plaques from the highest pCO_2_ treatment were on average 20% stronger than plaques produced in the lowest pCO_2_ treatment. (Fig. 3B, Table 1, lmem, *P* < 0.05). Although this trend is less evident in whole threads and plaques in the 25°C treatments, the effects of temperature and pCO_2_ did not interact significantly (Table 1, lmem, *P* = 0.6 and 0.2, respectively).

Additionally, threads produced at 25°C yielded at 60% lower force were 30% less extensible and 67% less stiff than threads produced at 10°C and 18°C (see Supplementary Figure S1 and Table S2; lmem, *P* < 0.001–0.05). As with thread breaking force, pCO_2_ had no effect on thread yield, extensibility, or stiffness (*P* = 0.51–0.7) and there was no significant interaction of temperature with pCO_2_ (*P* = 0.06–0.1). In summary, threads produced at 25°C were weaker, less stiff, did not yield in the distal region before failure, and were less extensible than those produced at 10°C or 18°C (Fig. 2C).

The tendency for threads to break in the proximal region increased with temperature (Fig. 3C, Pearson’s χ^2^, *P* < 0.05), from 40% at 10°C to 60% at 25°C. Distal breaks were infrequent and only occurred at 25°C, and pCO_2_ did not affect the breaking location of the threads (Fig. 3C, Pearson’s χ^2^*P* = 0.3). Paired comparisons of proximal and plaque strength within a thread indicate that the proximal region becomes relatively weaker with increased temperature (Fig. 3D; Kruskal–Wallis, *H* = 7.3, *P* < 0.05). Only threads from the 400 and 1200 pCO_2_ treatments were used for this analysis, due to limited sample size in the other treatments.

**Figure 4:**
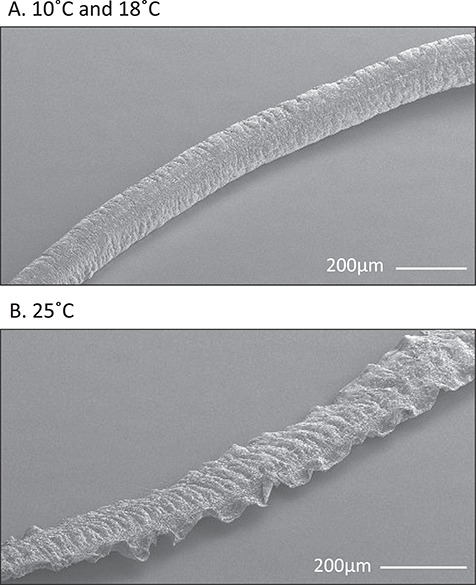
SEM images of the proximal region of a byssal thread produced at (A) 10°C and (B) 25°C. Threads produced at 25°C were typically flatter and malformed, with an added fringe of cuticle along the length of the thread.

The major axis of the proximal region did not differ among temperature treatments, but the minor axis was 27% thinner at 25°C than at 10°C (Table 2, lmem, *P* = 0.08 and *P* < 0.001, respectively). Proximal breaking stress was 47% lower at 25°C (Table 2, lmem *P* < 0.05). SEM images of proximal regions from 25°C suggest malformation in the outer cuticle, along the major axis (Fig. 4). This fringe was absent from all byssal threads formed at 10°C and 18°C.

**Table 2:** Increased temperature altered proximal region morphology and material strength. Compared to threads made at 10°C or 18°C, those made at 25°C showed no difference in major axis (lmem, *P* = 0.08) but were thinner in the minor axis (lmem, *P* < 0.001). Proximal region breaking stress, calculated as force to break divided by cross-sectional area, was reduced at 25°C (lmem, *P* < 0.05). Significantly different treatments are bolded; values are means ± SE, *n* = 35–48

**Temperature**	**Proximal Major Axis (mm)**	**Proximal Minor Axis (mm)**	**Proximal Stress (N mm** ^**−2**^ **)**
10°C	0.127 ± 0.005	0.036 ± 0.002	12.75 ± 1.59
18°C	0.138 ± 0.005	0.034 ± 0.002	15.31 ± 2.17
25°C	0.121 ± 0.005	**0.023 ± 0.002**	**6.73 ± 0.90**

**Figure 5:**
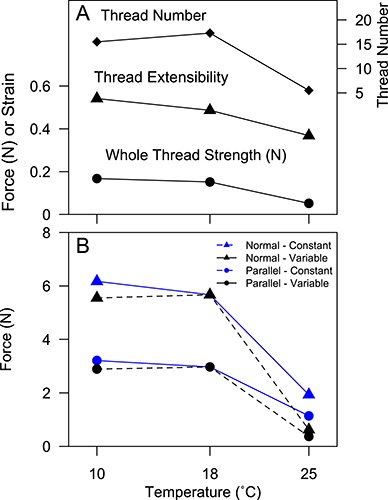
The force to dislodge a whole mussel (*M. trossulus*) as predicted by simulating a pull normal (triangles) or parallel (circles) to the substrate (Bell and Gosline, 1996), as a function of seawater temperature ranging 10–25°C. (A) Model inputs of thread number, thread extensibility, and whole thread strength were based on the results of this study and are reduced at 25°C. (B) Mussel attachment force was estimated from these inputs with either variable thread number (solid line; incorporating differences in thread quality and production among treatments) or constant thread number (dashed line; incorporating differences in thread quality only). Whole mussel attachment at 25°C decreased dramatically under all scenarios, especially when pulled normal to the substrate.

The strength of the entire byssus attachment was estimated using mean values of thread strength and extensibility for each temperature treatment (Fig. 5A). The model estimates for force to dislodge a mussel when pulled normal to the substrate were 68% lower at 25°C relative to 10°C and 18°C (Fig. 5B, constant thread production). When pulled parallel to the substrate, mussel attachment was 64% weaker at 25°C relative to 10°C and 18°C. Incorporating differences in thread production among treatments exacerbated the negative effects of high temperature, with 88% and 87% weaker attachment at 25°C in the normal and parallel models, respectively (Fig. 5B, variable thread production). The effects of pCO_2_ treatment did not affect these estimates substantially (data not shown).

## Discussion

A mussel’s defense against dislodgment by waves, currents, or predators relies on the byssal thread quality and quantity, both of which are dramatically compromised by elevated temperature in *M. trossulus*. Byssal threads were 60% weaker and 30% less extensible at 25°C, and these effects combine to lower the force that a mussel can resist by 64–68%, depending on the direction that the external load is applied. Mussels also produced fewer threads at 25°C, and when this result is incorporated into our attachment model, mussels are estimated to be up to 88% weaker than those at 10°C and 18°C. This magnitude of weakening exceeds that observed by Carrington *et al*. (2009) for *M. edulis*, where a ~ 50% decrease in attachment strength led to ~ 30% dislodgment mortality of the population.

Both morphological and material changes contribute to the observed decrease in thread breaking force at elevated temperature. Although this stressor weakened each region of the thread, the effects of elevated temperature were most pronounced in the proximal region, evidenced by the increased ratio of proximal to plaque breaks, from 1:1 to 3:2, and the disproportional decrease in proximal breaking force compared to plaque breaking force. The proximal region produced at 25°C was thinner and consequently had less load-bearing material. Moreover, the material itself was weaker, evidenced by a reduced breaking stress (breaking force normalized to size) at 25°C. While the nature of this material change in the proximal region was not explored in detail, we did observe malformed cuticle coatings, with a wide fringe along the side of the thread. Interestingly, this morphology is similar to that observed by Priemel *et al*. (2018) in threads induced to form by relaxing the foot with a KCl injection, suggesting that elevated temperature may inhibit the ability of *M. trossulus* to maintain pedal groove posture during thread formation. The presence of malformed byssal threads may be a useful visual tool to identify temperature stress in mussels, similar to the way that malformation has been used as a bioindicator of pCO_2_ stress in pteropods (Bednaršek *et al.*, 2012; Bednaršek *et al*., 2017).

Unlike the proximal region, the adhesive plaque was influenced by both elevated temperature and pCO_2_. Unexpectedly, elevated pCO_2_ increased plaque strength by 20% over 3 days, which runs contrary to the findings of O'Donnell *et al*. (2013), where plaque strength decreased by 40% in the same mussel species exposed over 30 days. Our follow-up studies comparing 3 versus 30 days of treatment exposure (Newcomb, 2015), however, did not indicate exposure time to be a modulating factor in plaque response nor were initial mussel condition indices, body weight, and reproductive condition able to explain these differences. However, one discrepancy between this study and O'Donnell *et al*. (2013) is that threads were harvested 3 days after being deposited by mussels, rather than 20 days, a difference that may explain the overall weaker maximum thread strength of the control treatment in this study. In aggregate, these results suggest some other factor, such as substrate fouling, nutrient levels, and/or some other seasonal trend as yet unidentified may modulate the susceptibility of byssal thread plaques to elevated pCO_2_ in this species (Waite and Broomell, 2012).

There is now considerable evidence that high CO_2_ (= low pH) inhibits the rate and final level of byssal thread plaque maturation over the first 12 days following deposition irrespective of any effect on the physiology of the mussel (George & Carrington, 2018; George *et al*., in press), which is consistent with the notion that the high seawater pH acts as a molecular trigger for byssus maturation following deposition (Waite, 2017). It is therefore likely that the differing responses to pCO_2_ between O'Donnell *et al*. (2013) and this study are due to effects on mussel physiology prior to thread deposition, similar to the altered expression of genes coding for foot proteins observed when pH decreases in *M. coruscus* by Zhao *et al*. (2017) and hypoxia as observed by Sui *et al*. (2017). Interestingly, no difference in size of plaques across treatments was observed in this study (data not shown) or O'Donnell *et al*. (2013), while plaque area was found to be reduced in the low pH treatment for both *M. coruscus* and *M. edulis* in Kong *et al*. (2019). The presence or absence of this difference in plaque size could serve as an indication of physiological stress and the susceptibility of a particular species to ocean acidification and would benefit from further study.

In addition to reducing byssal thread structural integrity, elevated temperature decreased thread production. Living at 25°C is energetically costly to *M. trossulus* (Braby & Somero, 2006); thus, energy reserves may be shifted away from byssus production towards other processes (Carrington *et al*., 2015; Sebens *et al*., 2018). Previous studies on *M. edulis* found that thread production increases as temperature ranges from 2°C to 18°C (Moeser & Carrington, 2006; Moeser *et al*., 2006; Young, 1985). The results reported here suggest that 25°C exceeds the point where temperature has a positive influence on thread production for *M. trossulus* and the temperature optimum for thread production in this species likely lies between 18°C and 25°C. Mussels used in this study were collected from a location where temperatures range as high as 25°C on warm summer days (Nishizaki & Carrington, 2014), suggesting that temperature effects on structural integrity could play a role in defining species distribution limits (Queirós *et al*., 2015; Kroeker *et al*., 2016). We note, however, that temperatures only reach 25°C on the hottest days in the summer, and it is worth exploring further how the interplay of warming level, duration, and frequency in relation to hydrodynamic loading determines when and where temperature will become too high to support viable mussel attachment. Such insights could inform the development of climate adaptation strategies that mitigate the impacts of warming temperatures on rocky coastlines, where mussels structure communities, as well as current and future mussel aquaculture locations (Ruckelshaus *et al*., 2013).

The results of this study highlight the importance of identifying which component of a byssal thread is affected by each stressor. Elevated temperature disproportionately influenced the thread proximal region while elevated pCO_2_ affected only the adhesive plaque. The two stressors are therefore not collocated and, as predicted, do not interact additively or synergistically. Instead, the stressors act on different but interconnected thread components, and it is the stressor with the greater deleterious effect, in this case elevated temperature, that dictates overall thread strength. Statistically, such a scenario should produce an antagonistic interaction between the two stressors, with an effect size exactly equal to that of the greater stressor. However, we did not observe any significant interaction terms for any of the response variables examined. A weak trend among the pCO_2_ treatments is nonetheless evident in whole thread and plaque strength at 10°C and 18°C, but not at 25°C; the lack of statistically significant interaction may simply be due to the small magnitude of the pCO_2_ effect and low statistical power.

Scaling up from individual byssal threads, it is the aggregate strength of the byssus structure that determines a mussel’s risk of dislodgement by a given force. A mussel can potentially overcome the perils associated with weak byssal threads by producing more of them, effectively redistributing the load to reduce the tension in each thread (Bell and Gosline, 1996). The findings of this study indicate that *M. trossulus* does not exhibit such a compensatory behavior with elevated temperature stress. Instead, reduced thread production exacerbated the deleterious effects of high temperature on individual thread strength and reduced byssus strength even further (Fig. 5). Similarly, Zhao *et al*. (2017) and Kong *et al*. (2019) report reduced thread strength and thread production under high pCO_2_ conditions in *M. coruscus* and *M. edulis*, respectively, and Dickey *et al*., 2019 report no effect in M. edulis. Taken together, these studies suggest that mussels do not behaviorally compensate for the delirious effects of environmental stressors on byssal thread integrity and that thread number is an important metric to measure, while investigating the effect of the environmental factors on mussel attachment and dislodgement risk.

Overall, this study indicates that the mussel *M. trossulus* is highly susceptible, in terms of weak attachment and associated dislodgment risk, to warming ocean conditions. A weaker attachment in a warmer ocean could cause a reduction in mussel density on rocky shores and aquaculture lines, which would have cascading ecological and economic impacts on coastal communities. Further study is needed to identify the threshold temperature for weak attachment relative to the temperatures encountered in natural habitats, and the concurrent hydrodynamic loading, to determine environmentally relevant safety factors and risk of failure (Johnson and Koehl, 1994; Carrington *et al*., 2009). Structural analysis provides a multiscale, predictive framework for guiding these efforts and for incorporating the effects of other potential stressors under current and future climate scenarios.

While the tethers of byssal threads present an intuitive parallel to chains and other structures that resist mechanical forces, the notion of weakest links can help explain the response of other physiological processes to environmental stressors. For example, the production capacity of a biosynthetic pathway is set by its rate-limiting step and the mass flow rate through a circulatory conduit is limited by the diameter of its narrowest region. From the biochemical to the ecological scale, investigation of responses to shifting environmental conditions should consider the isolated impacts on individual system components to understand mechanisms of impact. For instance, different species’ different degrees of susceptibility to environmental stressors mean that the aggregate ecosystem effect will depend on which system members are most susceptible (“the weakest links”) and the resilience of the system to these links breaking. Where multiple stressors co-occur (as they frequently do), especially in ways that produce heterogenous mosaics in space and time, these link-dependent effects may matter far more than the absolute magnitude of the impacts of single stressors on individual players. From a conservation standpoint, identifying the weakest links requires understanding the absolute susceptibility of individual players and the consequences of their failure.

## Supplementary Material

Newcomb_et_al_Supplementary_Tables_and_Figures_Revised_07132019_coz068
