# Supplementary material for: Only as strong as the weakest link: structural analysis of the combined effects of elevated temperature and pCO2 on mussel attachment
Source: Conserv Physiol. 2019 Oct 31;7(1):coz068. doi: 10.1093/conphys/coz068 (PMC6822540; doi:10.1093/conphys/coz068)
Supplement: Newcomb_et_al_Supplementary_Tables_and_Figures_Revised_07132019_coz068 [file newcomb_et_al_supplementary_tables_and_figures_revised_07132019_coz068.docx]

**Supplementary Tables and Figures**

| **pCO_2_ Target** |  | **Temperature** | | | **pH** | | | **Talk** | | | **DIC** | **pCO_2_** | | | **Mussel** |
| --- | --- | --- | --- | --- | --- | --- | --- | --- | --- | --- | --- | --- | --- | --- | --- |
| **µmol kg^-1^** | **Trial** | **˚C** | | | **Total Scale** | | | **µmol kg^-1^** | | | **µmol kg^-1^** | **µmol kg^-1^** | | | **Sample Size** |
| 2500 | 1 | 10.3 | ± | 0.4 | 7.22 | ± | 0.02 | 2093 | ± | 9.8 | 2168 | 2830 | ± | 54 | 8 |
| 1200 | 1 | 10.3 | ± | 0.6 | 7.50 | ± | 0.01 | 2093 | ± | 9.8 | 2092 | 1456 | ± | 26 | 7 |
| 750 | 1 | 10.1 | ± |  | 7.82 | ± |  | 2093 | ± | 9.8 | 2025 | 668 | ± | 11 | 7 |
| 400 | 1 | 9.9 | ± | 0.7 | 8.02 | ± | 0.00 | 2093 | ± | 9.8 | 1908 | 401 | ± | 7 | 8 |
| 2500 | 1 | 17.9 | ± | 0.3 | 7.32 | ± | 0.07 | 2093 | ± | 9.8 | 2142 | 2339 | ± | 75 | 8 |
| 1200 | 1 | 17.9 | ± | 0.8 | 7.63 | ± | 0.09 | 2093 | ± | 9.8 | 2081 | 1104 | ± | 41 | 8 |
| 750 | 1 | 17.9 | ± | 0.2 | 7.73 | ± | 0.07 | 2093 | ± | 9.8 | 2064 | 861 | ± | 26 | 5 |
| 400 | 1 | 18.1 | ± | 0.4 | 7.99 | ± | 0.00 | 2093 | ± | 9.8 | 1941 | 441 | ± | 7 | 6 |
| 2500 | 1 | 24.9 | ± | 0.1 | 7.28 | ± | 0.02 | 2093 | ± | 9.8 | 2111 | 2669 | ± | 51 | 6 |
| 1200 | 1 | 23.8 | ± | 0.3 | 7.60 | ± | 0.05 | 2093 | ± | 9.8 | 2022 | 1216 | ± | 31 | 5 |
| 750 | 1 | 25.0 | ± | 0.1 | 7.77 | ± | 0.00 | 2093 | ± | 9.8 | 1953 | 793 | ± | 14 | 4 |
| 400 | 1 | 24.7 | ± | 0.2 | 8.00 | ± | 0.01 | 2093 | ± | 9.8 | 1867 | 430 | ± | 7 | 5 |
| 2500 | 2 | 10.0 | ± | 0.1 | 7.31 | ± | 0.44 | 2085 | ± | 6.8 | 2186 | 2274 | ± | 385 | 6 |
| 1200 | 2 | 10.1 | ± |  | 7.44 | ± |  | 2085 | ± | 6.8 | 2113 | 1652 | ± | 24 | 7 |
| 750 | 2 | 10.0 | ± | 0.1 | 7.72 | ± | 0.04 | 2085 | ± | 6.8 | 2013 | 849 | ± | 17 | 6 |
| 400 | 2 | 10.1 | ± | 0.1 | 8.20 | ± | 0.07 | 2085 | ± | 6.8 | 1882 | 248 | ± | 7 | 7 |
| 2500 | 2 | 17.9 | ± | 0.0 | 7.29 | ± | 0.01 | 2085 | ± | 6.8 | 2259 | 2503 | ± | 38 | 8 |
| 1200 | 2 | 17.9 | ± | 0.1 | 7.58 | ± | 0.02 | 2085 | ± | 6.8 | 2040 | 1243 | ± | 20 | 5 |
| 750 | 2 | 17.9 | ± | 0.0 | 7.77 | ± | 0.01 | 2085 | ± | 6.8 | 1985 | 775 | ± | 11 | 6 |
| 400 | 2 | 17.8 | ± | 0.1 | 8.23 | ± | 0.06 | 2085 | ± | 6.8 | 1898 | 227 | ± | 6 | 7 |
| 2500 | 2 | 24.9 | ± | 0.0 | 7.32 | ± | 0.01 | 2085 | ± | 6.8 | 2123 | 2414 | ± | 37 | 2 |
| 1200 | 2 | 24.9 | ± | 0.0 | 7.53 | ± | 0.05 | 2085 | ± | 6.8 | 2077 | 1447 | ± | 34 | 3 |
| 750 | 2 | 24.9 | ± | 0.0 | 7.81 | ± | 0.06 | 2085 | ± | 6.8 | 1962 | 712 | ± | 18 | 2 |
| 400 | 2 | 24.9 | ± | 0.0 | 8.14 | ± | 0.01 | 2085 | ± | 6.8 | 1821 | 290 | ± | 4 | 4 |
| 2500 | 3 | 24.9 | ± | 0.0 | 7.31 | ± | 0.00 | 2085 | ± | 8.4 | 2128 | 2466 | ± | 40 | 6 |
| 1200 | 3 | 24.9 | ± | 0.3 | 7.58 | ± | 0.00 | 2085 | ± | 8.4 | 2115 | 1274 | ± | 20 | 6 |
| 400 | 3 | 23.8 | ± | 2.1 | 8.12 | ± | 0.10 | 2085 | ± | 8.4 | 1807 | 306 | ± | 12 | 8 |

Supplementary Table S1. Water conditions and mussel sample size in the different treatments in all trials. Temperature and pH are mean ± SD from monitoring every minute in the water reservoirs. pH is reported on the total scale. Total alkalinity (Talk) represents the mean of 7-9 samples taken from randomly selected chambers in different reservoirs and is reported for all treatments across a trial. Dissolved inorganic carbon (DIC) was measured once in a reservoir. pCO_2_ was calculated from the average pH and Talk with error propagated according to Ellison et al. (2000) using sensitivity coefficients from Dickson and Riley (1978). The few missing error terms are due to equipment malfunction.

Stiffness (N mm^-2^)

Supplementary Figure S1. Byssal thread yield force (A), extensibility (B) and stiffness (C) as a function of pCO_2_ and temperature. Error bars represent ± SEM; see Supplementary Table S2 for a summary of statistical analyses. All thread properties varied significantly with temperature (lmem, p < 0.001); threads at 25˚C yielded at a lower force, were less extensible and less stiff than threads at 10˚C and 18˚C (Tukey HSD, p < 0.05). For all three response variables, the effect of pCO_2_ and its interaction with temperature was not significant, lmem, p = 0.051-0.7 and 0.06-0.1, respectively). Table S2. Summary of statistical analyses of elevated temperature and pCO_2_ on byssal thread yield force, extensibility and stiffness. Linear mixed effect models were run with temperature and pCO_2_ as fixed factors and trial as a random factor on each of the dependent variables; see Table S1 for N.

|  |  |  | **Distal**  **Yield (N)** | | **Thread Extensibility** | **Thread**  **Stiffness (N mm^-2^)** |
| --- | --- | --- | --- | --- | --- | --- |
| random effect of trial | | stdev | 0.000 | | 0.01 | 0.02 |
| ANOVA  on lmem | Temperature | χ^2^ | | 40.6 | 8.2 | 78.0 |
|  |  | d.f. | | 2 | 2 | 2 |
|  |  | p | | **< 0.001** | **< 0.05** | **< 0.001** |
|  | pCO_2_ | χ^2^ | | 1.3 | 2.0 | 7.8 |
|  |  | d.f. | | 3 | 3 | 3 |
|  |  | p | | 0.7 | 0.6 | 0.051 |
|  | Temperature*pCO_2_ | χ^2^ | | 11.3 | 9.6 | 11.7 |
|  |  | d.f. | | 6 | 6 | 6 |
|  |  | p | | 0.07 | 0.1 | 0.06 |
| Tukey HSD | 10˚C |  | | a | a | a |
|  | 18˚C |  | | a | ab | a |
|  | 25˚C |  | | b | b | b |
|  |  |  | |  |  |  |
